# AITSO: A Tool for Spatial Optimization Based on Artificial Immune Systems

**DOI:** 10.1155/2015/549832

**Published:** 2015-01-05

**Authors:** Xiang Zhao, Yaolin Liu, Dianfeng Liu, Xiaoya Ma

**Affiliations:** ^1^School of Resource and Environmental Science, Wuhan University, Wuhan 430079, China; ^2^Key Laboratory of Geographic Information System, Ministry of Education, Wuhan University, Wuhan 430079, China

## Abstract

A great challenge facing geocomputation and spatial analysis is spatial optimization, given that it involves various high-dimensional, nonlinear, and complicated relationships. Many efforts have been made with regard to this specific issue, and the strong ability of artificial immune system algorithms has been proven in previous studies. However, user-friendly professional software is still unavailable, which is a great impediment to the popularity of artificial immune systems. This paper describes a free, universal tool, named AITSO, which is capable of solving various optimization problems. It provides a series of standard application programming interfaces (APIs) which can (1) assist researchers in the development of their own problem-specific application plugins to solve practical problems and (2) allow the implementation of some advanced immune operators into the platform to improve the performance of an algorithm. As an integrated, flexible, and convenient tool, AITSO contributes to knowledge sharing and practical problem solving. It is therefore believed that it will advance the development and popularity of spatial optimization in geocomputation and spatial analysis.

## 1. Introduction

The process during which spatial entities achieve the optimal status under certain constraints is referred to as spatial optimization [1]. It has recently become one of the critical issues in geoscience research since most practical spatial planning problems can be regarded as a typical spatial optimization process, such as spatial sampling optimization [2], location and allocation problems [3, 4], path optimization problems [5, 6], land-use optimization [7, 8], and protected natural areas zoning [9]. Spatial optimization generally involves various high-dimensional, nonlinear, and complex relationships. The majority of the spatial analyses or geostatistical models provided by geographical information systems (GIS), however, are limited in their ability to address such problems. Under such circumstances, artificial intelligence (AI) has been advocated and has proven to be promising in spatial optimization.

Up to now, a number of AI methods have been proposed, such as genetic algorithms (GA) [10–12], simulated annealing (SA) [13, 14], ant colony optimization (ACO) [15, 16], particle swarm optimization (PSO) [12, 17], tabu search [18], and artificial immune system (AIS) [9, 19]. Among these artificial intelligence algorithms, AIS has proven to be reliable and efficient in various cases, such as remote sensing imagery processing [20], spatial data mining [21], routing problems [22], location problems [23], and land-use dynamics [9, 19, 24].

AIS can be defined as intelligent and adaptive computational systems inspired by theoretical immunology principles and mechanisms in order to solve real-world problems [25, 26]. Although the previous studies have greatly contributed to AIS development, the algorithms described were too problem-specific, since scholars merely focused on one specific problem. Few reports have been released that have proposed a unified algorithm framework or developed an integrated computational platform. Therefore, this paper aims to address these concerns by developing a universal tool for spatial optimization. Our specific objective is to develop an integrated, extensible, and customizable computational framework called AITSO that can be utilized for solving different optimization problems.

## 2. Available Tools/Libraries for Solving Optimization Problems

During the past decade, researchers have developed many spatial decision support systems for use in solving spatial optimization problems [27, 28], and most of them are problem-specific. There are, however, few universal software packages that have been specifically designed for solving spatial optimization problems. On the other hand, many software or libraries have implemented AI algorithms, which have been widely used in solving optimization problems. Therefore, it has been necessary to gather some details about the existing AI tools in order to get some useful experience to help with the development of AITSO. The main features of the best known and most popular intelligence optimization platforms or libraries are listed in Table 1.

Among these platforms or libraries, GeoSOS (Geographical Simulation and Optimization System, website: http://www.geosimulation.cn/) is the only one that has been designed for solving geographical simulation and spatial optimization problems [29]. It was developed based on the open-source GIS MapWindow components with basic GIS functions such as the handling and displaying of spatial data. GeoSOS has integrated many intelligence models, including cellular automata (CA), agent-based models (ABMs), and swarm intelligence models (SIMs). It is a powerful tool for solving urban simulation and land-use planning problems. However, the optimization models implemented in GeoSOS were still too problem-specific. Consequently, in some cases, it might be incapable of meeting the requirements of modeling various complicated spatial optimization problems.

Another universal tool for solving optimization problems is OAT (the Optimization Algorithm Toolkit, website: http://optalgtoolkit.sourceforge.net/). OAT is an open-source software package written in Java that integrates many of the computational intelligence optimization algorithms, including GA, ACO, SA, PSO, and CSA. It was developed based on a very flexible architecture to meet the demands of users with different roles, including interested amateurs, software developers, and research scientists. Users can adapt the universal tool to their specific problem in two ways in this architecture: (1) modify the source code of OAT to make it suitable for the problem or (2) use OAT as a library and develop new application plugins based on the abstract classes or user interfaces defined by OAT [30, 31]. Generally, OAT has provided an open, universal, and integrated platform for solving optimization problems and comes with a user-friendly interface. However, OAT uses “domains” to organize problems which are independent of each other. That means users still have to rewrite the stopping conditions and immune operators for their optimization problems. Therefore, the architecture of OAT is not conducive to the reusing and sharing of immune operators since the algorithms are designed coupled with the optimization problems. Nevertheless, it has provided some useful experience for us that has helped with the design of AITSO.

EO (Evolutionary Objects, website: http://eodev.sourceforge.net/) is another open-source library for solving optimization problems. EO has built a generic algorithm framework and implemented a well-designed library, which was written in ANSI-C++ and is based upon template technology. In the algorithm framework of EO, the algorithms are divided into several operators, and the operators are further categorized into two subsets: “evolution engine” and “representation-specific operators.” The former is only concerned with the fitness of the individuals, and it is totally independent of the specific problems. The latter is coupled with specific problems and depends on some particular data structures [32]. This design makes it possible to solve different problems in a unified algorithm framework, and it is conducive to code reusing. Researchers can adapt the universal algorithms to their specific problems by defining their own data structures and implementing the “representation-specific operators,” and the “evolution engine” does not require redevelopment. However, EO does not offer a user interface, and all these functions are provided in the form of programming libraries. Generally, EO can be considered as a semifinished product, and it is mainly designed for developers who need to integrate the library into their own decision support system to solve a particular problem.

The GAtool is one of the tools in MATLAB's global optimization toolbox. It has provided researchers with the ability to apply genetic algorithm techniques to optimization problems in the MATLAB environment. The GAtool contains many predefined genetic operators, and users can also define their own operators by writing some MATLAB scripts. As this framework is a tool of MATLAB, it means that GAtool requires MATLAB to be installed, and users have to get a license to run the solver. Furthermore, the MATLAB software does not support the handling and displaying of spatial data, and the integration of GIS functions into MATLAB is usually challenging.

From the above review of the well-known intelligence optimization tools, we can conclude that most of the available tools or libraries for solving optimization problems still cannot directly meet the demands of spatial optimization. A new integrated, universal, and extensible tool is needed to advance the research into spatial optimization and to improve the ability of decision support for practical applications.

## 3. Design and Implementation of the Tool

### 3.1. System Requirements Analysis

#### 3.1.1. Spatial Modelling Needs Analysis

To develop a general purpose tool, it is essential to analyze the fundamental requirements of spatial optimization modelling. Like general optimization problems, spatial optimization problems can also be described as follows:

(1) Max⁡    f(x)Subject  to: [C.E.]      [B.C.],

where *f*(*x*) is the objective functions of the optimization problems, and the constraints are conducted by “Subject to.” “[C.E.]” is the condition equations, usually composed of a series of equations or inequalities, and all the solutions should meet these constraints. “[B.C.]” is the boundary conditions of the optimization problem, which is used to specify the domain of the decision variables.

However, compared with nonspatial optimization problems, spatial optimization problems are more complicated and more particular.

- The representation of spatial optimization problems is more complicated and more particular than nonspatial problems. When using immune algorithms to solve an optimization problem, the first step of the process is to encode the solution of the problem into “artificial antibodies” by using an encoding strategy (binary, real number, etc.). Then, an antibody corresponds to a solution to the problem, and each spatial entity or variable of the solution is represented as a “gene” in the antibody. However, the data structures, which are used to represent the location and status of the entities or variables, are usually more complicated than in nonspatial problems. Therefore, researchers usually have to redesign the encoding strategy and data structures for each specific problem.
- The objective functions of optimization problems are distinct from each other, and most spatial optimization problems are multiobjective problems. When solving a spatial problem in geospatial modelling, the design of the objective functions usually has to incorporate the ecological context and social and economic criteria under some predetermined scenarios.
- The constraints of spatial optimization problems might include some complex spatial constraints such as topological constraints. Furthermore, the mutation operation of antibodies is not usually completely random but is conducted with some specific domain knowledge. For example, when solving land-use spatial allocation problems, the “mutation” operation means to change the land-use type of some parcels. Therefore, this operation should be implemented based on the physical and socioeconomic properties of the land. Furthermore, the properties of the neighboring parcels should also be considered.
- Solving spatial optimization problems requires customization of the simple immune algorithms. Usually, researchers have to customize the basic immune algorithms to improve the performance of the algorithms, especially in solving complex spatial problems. In most cases, the immune algorithms can be improved from the following two aspects: (i) hybridization with other global optimization search algorithms such as GA or ACO to avoid the algorithm falling into a local optimum and (ii) incorporation of a local search mechanism to accelerate the convergence of the immune algorithm.
- The inputs/outputs of different spatial optimization problems are distinct in content, structure, and format.

In summary, to determine the appropriate encoding strategy, objective functions, constraints, and inputs/outputs of the artificial immune model for spatial optimization is the most important problem to the users. The steps or operators mentioned above are problem-specific and should be integrated into AITSO as standard application interfaces. Furthermore, the process of the algorithm framework should be extensible to meet the customization demands of the algorithm.

#### 3.1.2. User Needs Analysis

As a universal tool, we assume that the users who might use this tool can be divided into the following classes based on their background knowledge and skills.

*Immune Algorithm Researchers*. These users are familiar with immune algorithms and they are interested in improving the performance of algorithms. These users can use the programming interfaces provided by AITSO to develop advanced immune operators. Once a new operator is developed and integrated into AITSO, it can then be used to solve any optimization problems. 

*Spatial Optimization Problem Researchers*. These users have rich experience in modeling and solving optimization problems. However, they might not be familiar with immune algorithms. For these users, the only thing they have to do is to focus on modeling the specific problems based on the application programming interfaces provided by AITSO. 

*Decision Makers*. This type of users might not have training in programming. However, they are interested in solving practical spatial optimization problems by the use of immune algorithms. For these users, they can solve their problems by using the applications and the immune algorithms developed by the former two types of users.

### 3.2. Software Design and Key Technologies of the Tool

#### 3.2.1. Common Spatial Optimization Model Design

A unified spatial optimization model is a prerequisite for the establishment of a universal tool. Such a model must meet two basic requirements. For one thing, the model must be extensible as, in practice, algorithms are usually revised and improved to solve specific problems. The model should therefore be able to integrate different algorithms and to customize the process and operators. For another, the algorithms or operators that are integrated into the framework must be reusable. When a new algorithm or operator is developed, it should be able to be used in other cases.

We referred to the principle proposed by Keijzer et al. [32]. Here, the immune algorithms are decomposed into two parts: one includes the common immune mechanism, and the other is problem-specific. Two steps are included, as follows [33, 34].

**Figure 1:**
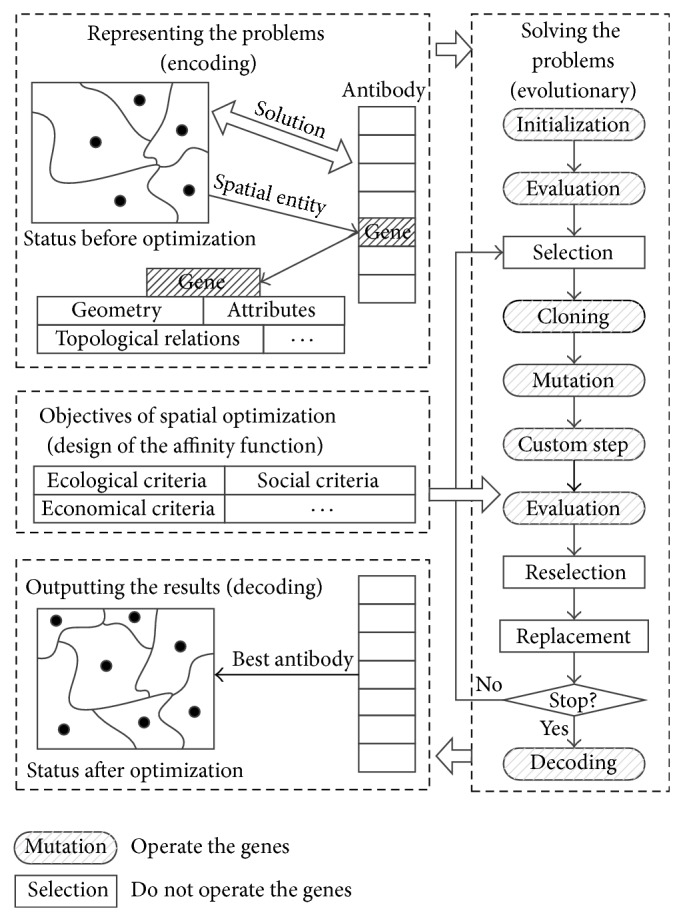
The common artificial immune model for spatial optimization.

1. As shown in Figure 1, most of the artificial immune system based optimization algorithms can be decomposed into eight steps: initialization, evaluation, selection, cloning, mutation, reselection, replacement, and decoding, and the details of the algorithm can be found in the previous studies [34, 35]. In some cases, to improve the performance of the algorithm, some custom steps (e.g., crossover and hill climbing algorithm) can be added to the process. Each of the steps can be considered as an evolutionary unit, which is used to carry out some special evolutionary task. Based on the basic immune principles, researchers can implement several particular versions of “operators” to accomplish a specific immune evolutionary task. Therefore, users can combine the different versions of operators to form a new artificial immune algorithm.
2. All the immune operators mentioned above can be divided into two categories, according to whether or not the operator has to operate the genes of an antibody. Since the data structure of genes is usually problem-specific, the operators which need to change the genes have to know the data structure of the genes. The selection operator, reselection operator, replacement operator, and the stopping criteria are only concerned with the affinity of the antibodies and are totally independent of the specific problems. Therefore, these operators can be considered as common immune operators and can be used to solve different problems. Other operators, such as initialization operators, evaluation operators, clonal operators, mutation operators, and decoding operators, are problem-specific and have to be redesigned for particular problems.

A critical shortcoming of this design is that users still have to redesign the problem-specific operators when the data structures of the genes are changed. For instance, the mutation operators, which have to change the values or status of the genes, are dependent on the exact data structures of the genes. However, the functions used to operate the genes are dependent on some particular data structures. Nevertheless, the immune principle used to calculate the mutation rate might be the same: the higher the affinity, the smaller the mutation rate [35]. Therefore, we expect that these data structure-dependent operators, with the same immune principles, can also be reused. In order to achieve this goal, we need more in-depth research.

**Figure 2:**
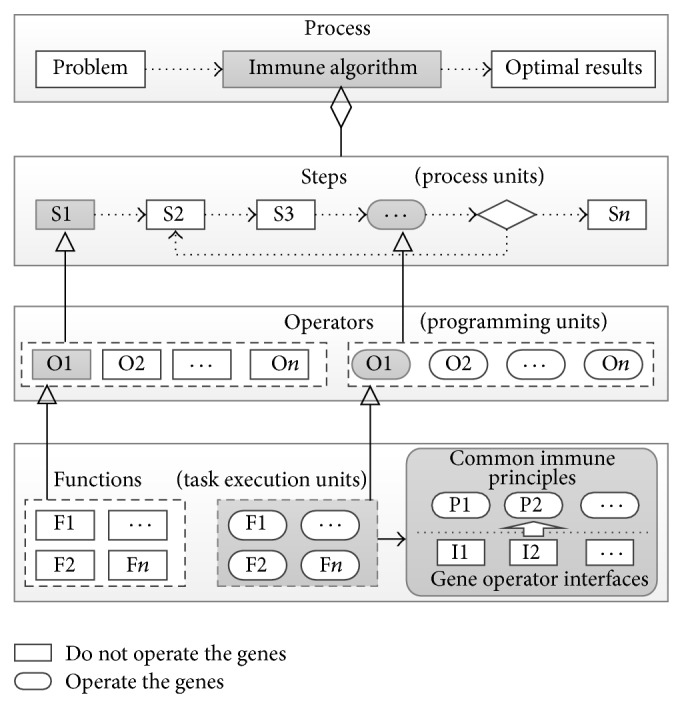
The hierarchical model of the unified optimization algorithm framework.

As shown in Figure 2, the process of an immune algorithm can be divided into several steps, which can be seen as logical process units. Users can add some custom steps to the process and adjust the execution order of each step to accomplish the customization of the algorithm. Every developer can develop operator plugins and release them to AITSO to share their work. Hence, the operators can be considered as the programming units. Furthermore, we hope that every operator class can be used to package a group of functions which have the same immune principles. As a result, the immune evolutionary tasks are finally assigned to the functions. In this sense, the functions are the real task execution units.

The functions that do not have to operate the genes can be applied to different problems directly, and the reuse of the problem-specific functions is implemented by the use of object-oriented technology. As stated above, the functions that are used to operate the genes can be encapsulated into an abstract class or interface to provide the immune operators or functions with standard function interfaces to access the genes.

#### 3.2.2. Software Architecture Design

**Figure 3:**
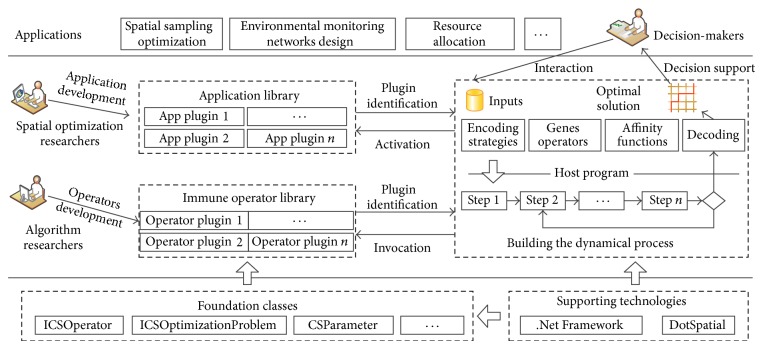
The components of AITSO and their relationships.

One of the most important purposes of AITSO is to bridge the gaps between immune algorithm researchers, spatial optimization problem researchers, and decision makers. To achieve this goal and to meet the different demands from these users, AITSO has adopted a very flexible architecture based on a “plugin host” structure to build the platform. As shown in Figure 3, AITSO is composed of four key components: the foundation class library, the immune operator library, the application library, and the host program. The function and the relationships between the four components are described as in Figure 3.

The foundation class library comprises the definition of the fundamental abstract classes, interfaces, enumerations, and some ancillary classes. These abstract classes and interfaces define the primary properties and behavior of the immune operators, antibodies, and optimization problems. Once a class implements all the properties and functions of the “ICSOperator” interface, the host program will identify it as a validated operator class. Similarly, the “ICSOptimizationProblem” interface is designed for developing application plugins that are used for solving specific spatial optimization problems. The abstract class “CSAntibody” is used for extending the data structures of the antibodies, and the parameters' information is stored by using the “CSParameters” class.

All the immune operators are stored in a folder named “Operators” in the form of dynamic link library (DLL) files. Algorithm researchers or anybody who wants to improve the performance of the algorithms can use the “ICSOperator” interface to develop a novel operator plugin. When the host program runs, the host program will search the DLL files contained in this folder and extract all the classes inherited from the “ICSOperator.” After that, an instance of the operator class will be activated by the reflection technology provided by the  .NET Framework. Finally, the information about the operator class, such as the name, description, functions, and the parameters, can also be extracted and displayed to users through the GUI.

Similar to the “immune operator library,” the “application library” is a folder named “Problems,” which stores many application plugins. An application plugin is a DLL file which has encapsulated one or more spatial optimization problem classes inherited from “ICSOptimizationProblem.” Once an application class is activated, the host program can obtain the description and parameters from the instance of the class and show them to the final users.

The major functionality of the host program is to link the decision makers and underlying libraries, which include the application library and immune operator library. Therefore, the host program plays two crucial roles in the whole platform. One is that it provides the user with a friendly GUI. The application plugins and operator plugins are identified based on the standard interfaces, and then the basic information (name, description, parameters, etc.) of the plugins is extracted and displayed to the user via the host's GUI. Thus, decision makers can accomplish their work interactively, for example, choosing the corresponding application plugin, customizing the immune algorithm, setting the parameters, and configuring the inputs/outputs of the problem. The other role of the host program is that it builds the process of the model and carries out the optimization tasks. Once the user has completely defined their problem and optimization model, the host program will activate the instance of the application and operator classes specified by the user. After that, the model will be constructed dynamically, according to the user's configuration, to accomplish the computation task.

**Figure 4:**
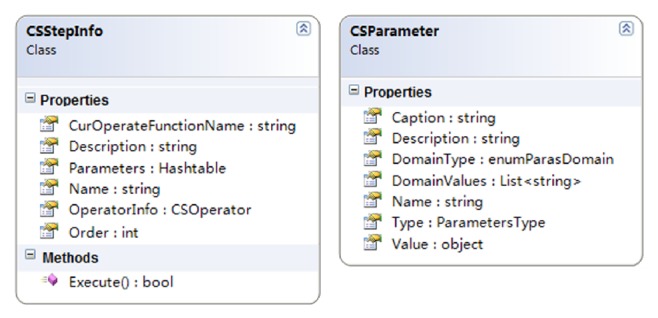
The class schema diagram of “CSStepInfo” and “CSParameter.”

The key technology for customizing the spatial optimization model is the design of the “CSStepInfo” class (see Figure 4). Each instance of CSStepInfo records the model steps information, including the execution order in the model's process, the operator class used for computation, function name, and the parameters' information gathered from the GUI. Therefore, the process of the model, as defined by the user, can be stored as an object array of the CSStepInfo in the host program. Once the algorithm is started, it can complete an iteration process by calling the “execute” member function of each step objects stored in the array.

Furthermore, the “CSParameter” class was designed to describe and manage the parameters of the model (see Figure 4). All the parameters of the model should be published, along with their metadata and description, since this is essential to users when configuring the proper values for the parameters. Furthermore, if the values of a parameter are constrained to some special values or particular value ranges, the developers can also write these enumerations or domain values into the “ParasDomain” property of the class. Therefore, the host program can generate the proper input controls (such as ComboBox and NumericUpDown) for users, so as to guarantee that the input values are validated.

#### 3.2.3. Spatial Optimization Applications Development Techniques

**Figure 5:**
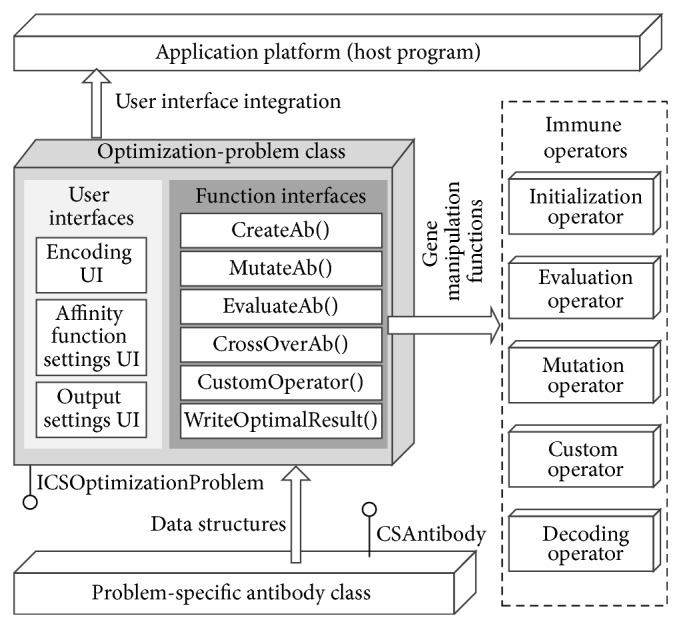
Structure of the optimization problem class.

**Figure 6:**
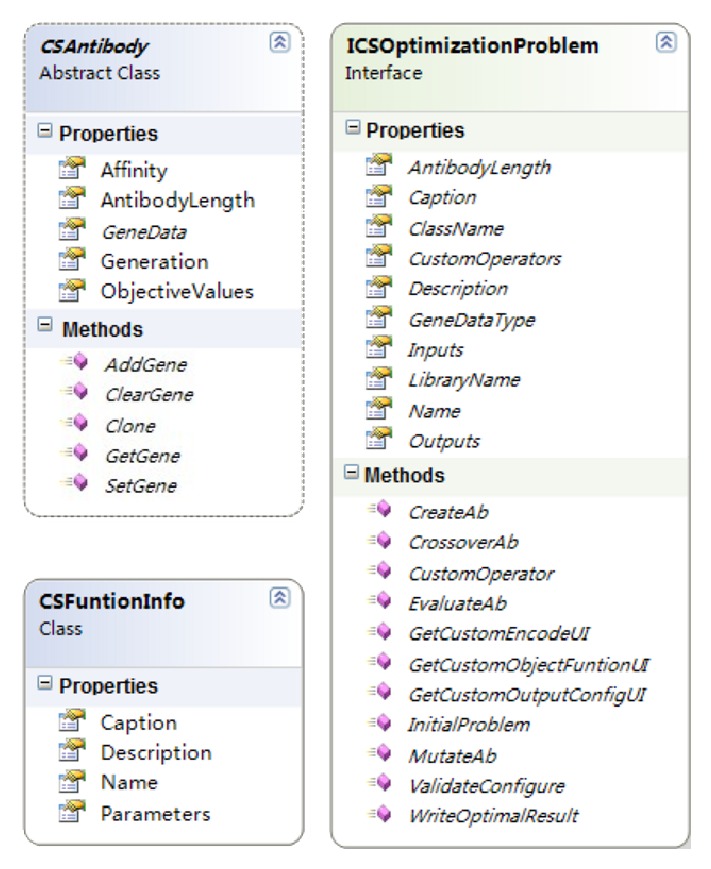
The schema diagram of the primary classes used for developing application plugins.

As stated in Section 3.1, developers usually have to redesign the encoding strategy, objective function, constraints, inputs, outputs, and the data structure of genes for each spatial optimization problem. On the other hand, it is advantageous that all the immune operators can be reused, and the development of applications is simple enough. Therefore, two interface/abstract classes were designed to encapsulate all these particular requirements. The architecture of a typical application class and its relationship with the other components in AITSO are illustrated in Figure 5, and the schema diagram of the primary classes is shown in Figure 6.

The antibody is the fundamental operating unit in immune algorithms, and it represents a solution to the optimization problem. The abstract class “CSAntibody” defines the basic properties and behaviors of the general antibodies. Once a new encoding strategy is proposed, developers can create a problem-specific antibody class, inherited from “CSAntibody,” and design the data structure for the genes. In addition, the basic gene manipulation functions, “AddGene,” “ClearGenes,” “GetGene,” “SetGene,” and “Clone,” should also be implemented. Among these functions, the functionality of “Clone” is to get an identical antibody to its parent, and it can be employed by the clonal operators.

The “ICSOptimizationProblem” interface is composed of two types of interfaces that are termed “user interfaces” and “function interfaces.” The “user interfaces” in the “ICSOptimizationProblem” are a group of  .NET Framework “user controls.” The interfaces are designed for the end-user, since they can provide the user with friendly wizards to configure the parameters, constraints, inputs, and outputs. Once an application class is activated, the “user interfaces” implemented in the “ICSOptimizationProblem” class will be instantiated and integrated into the host program GUI framework so as to gather parameters settings and so forth.

The “function interfaces” include six standard function prototypes declared in “ICSOptimizationProblem.” They are used to integrate the problem-specific gene-based operations and provide the immune operators with interfaces for assessing the genes of an antibody. Among the six function interfaces, the “CreateAb” function is used to create a problem-specific antibody object, and it can be invoked by the initialization operators; the “MutateAb” function is used to change the values or status of the genes according to the user-specified mutation rate under the constraints or domain knowledge; and the objective functions used to calculate the objective values and the affinity of the antibodies are packaged into “EvaluateAb.” Some complex methods such as the paretodominance approach for dealing with multiobjective problems and the penalty functions for handling the constraints can also be integrated into “EvaluateAb,” as needed. Furthermore, as the crossover operator has been widely used in evolutionary algorithms, the “CrossOverAb” function is defined to integrate the crossover mechanism to improve the performance of the immune algorithm. Once an optimization task is finished, the decoding operators can call the “WriteOptimalResult” function to save the optimal solution into the result file. The host program will also load the results to a mapping window to visualize the optimal solution and draw a convergence curve to analyze the performance of the algorithm.

Developers can also package some other operators (local search algorithms, etc.) into the algorithm by implementing the “CustomOperator” function. For example, when solving the traveling salesman problem (TSP), a 2-opt or k-opt local search can be employed to improve the performance of the algorithm [36]. We can implement all these algorithms into the class and call them through the “CustomOperator” function.

#### 3.2.4. Artificial Immune Algorithms Development Techniques

**Figure 7:**
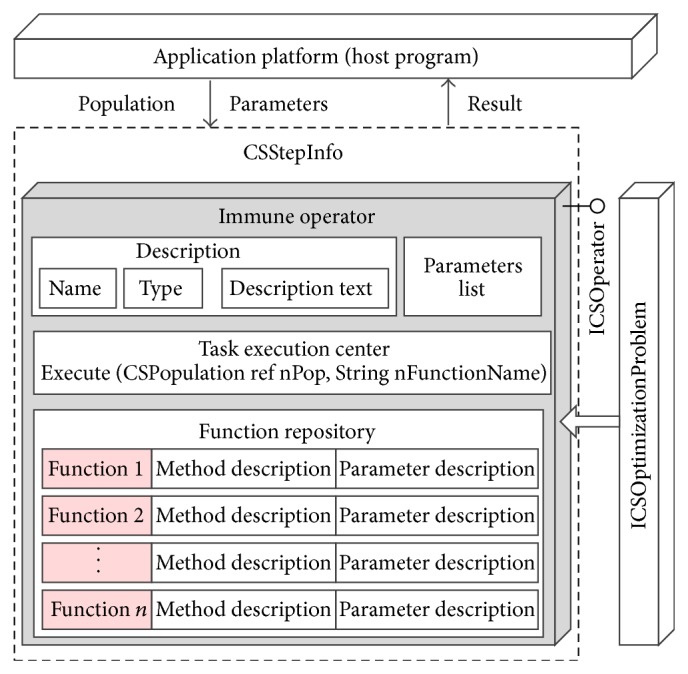
Structure of the operator class.

In order to get a general understanding of how the immune operator plugins work, the overall architecture of a typical operator class and its relationship to the other components in AITSO are illustrated in Figure 7. A typical operator class comprises four key parts, including description, parameter list, function repository, and the task execution center. For each operator class, the descriptive information provides the user with some essential information about the operator class, which can be identified and represented by the host program. The operator type information tags the execution stage (selection, mutation, replacement, etc.) of the operator class. Therefore, the host program can match the available operators for each algorithm step, according to the “Type” property of the operators. Developers can also provide the user with some useful hints by writing information into the “description text” when releasing their plugins.

The “function repository” usually comprises one or several functions implementing various immune strategies. It allows developers to package a group function into one operator class. For example, as stated in Section 3, the mutation rate is determined based on the affinity of each antibody: the higher the affinity, the smaller the mutation rate. This means that developers can design any formula to calculate the mutation rate, as long as the formation can implement the above principle, and package them into the same operator class. Nevertheless, all functions should be released together with their parameters and descriptive information, so that users can get a grasp of the methods to use, the functionality of the function, and can configure validating parameters for the functions.  Algorithm 1 is a simple example of implementation of the mutation function. In this example, the mutation rate is calculated by using the following formula [37]:

(2) $$\begin{array}{c} F^{\prime }\left(ab_{i}\right)=\frac{F\left(ab_{i}\right)-\min\left(F\left(ab_{i}\right)\right)}{\max\left(F\left(ab_{i}\right)\right)-\min\left(F\left(ab_{i}\right)\right)},\;i=1,2,\ldots ,N_{c},\\ P_{m}=\frac{\exp\left(-2*F^{\prime }\left(ab_{i}\right)\right)}{t}, \end{array}$$

where  *F*(*ab*_*i*_)  is the affinity of an antibody, *N*_*c*_ is the number of antibodies in the new population, and *F*′(*ab*_*i*_) is the normalized affinity of the antibody; *P*_*m*_ is the mutation rate of an antibody; 2 is the empirical value to control the decay; and *t* is the current number of iterations.

Once an operator class is activated to carry out the evolution tasks, the population of antibodies and the parameters will be sent to the instance of the operator from the host program. Then, the host program will call the “execute” method of the class to accomplish the specific task. There are two parameters of the “execute” module: the population is passed by using the “CSPopulation” parameter, and the function used to execute the calculation task is specified by the “function name” parameter. If the functions have to operate the genes of the antibody, the standard gene operation interfaces provided by “ICSOptimizationProblem” are employed.

### 3.3. Main GUI and Features of the Tool

**Figure 8:**
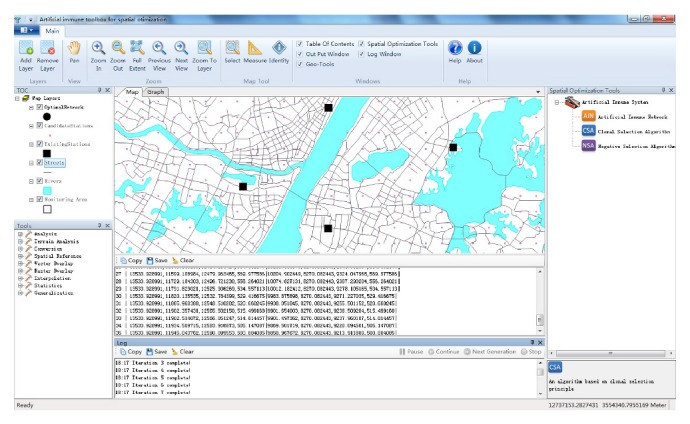
Main GUI of AITSO.

**Figure 9:**
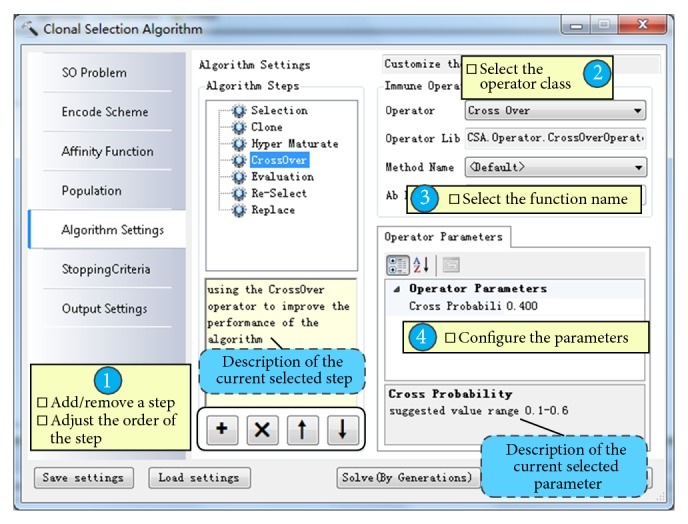
Steps for customizing the spatial optimization model.

AITSO was developed based on the open-source DotSpatial GIS components (http://dotspatial.codeplex.com/), which were developed based on the C# programming language and have been widely used in many fields [29, 38]. The C# 4.0 programming language was chosen to develop AITSO, as with DotSpatial. The technology of assembly reflection provided by the  .NET Framework is a very powerful and convenient tool for building operators or application plugins. Furthermore, parallel programming technology has been introduced to the  .NET Framework 4.0, which is a very useful tool for promoting the performance of computation. The main graphical user interface (GUI) of AITSO is shown in Figure 8, and the GUI for customizing the algorithm is shown in Figure 9.

As shown in Figure 8, AITSO has a main interface that is similar to most desktop GIS programs, plus the addition of tools associated with data visualization and analysis. The basic GIS functionality of AITSO is powered by the open-source DotSpatial GIS components. The artificial immune system algorithms which are designed for spatial optimization are organized as a tree list in the right dockable window. Once an optimization task is started, the log and result will be output in the corresponding windows at the bottom in real time. Furthermore, users can suspend or terminate the task when the algorithm is stagnated.

Figure 9 shows the main steps of the customization of the immune algorithms in the system: (i) specify a step, which needs to specify an operator, from the predefined steps or a custom step added by a user; (ii) the details of the matching operator classes which can be used in the current selected step will be listed in the upper-right ComboBox control; (iii) the names of the functions, which are packaged in the selected operator class, will be listed in the following ComboBox control; and (iv) once the function name is specified, the parameters of the function will be added to the property grid in the lower-right corner of the dialog, so that the user can set the values for these parameters.

## 4. Application Examples

### 4.1. The Traveling Salesman Problem

To test the reusability and the extensibility of the platform, we adopted the traveling salesman problem (TSP) as a benchmark testing application. TSP is a typical NP-hard combinatorial optimization problem [39, 40]. It involves all aspects of combinatorial optimization and has served as a benchmark problem for testing the performance of algorithms [41].

To solve this problem in AITSO, we developed an application plugin which integrates two key classes: “CSTSPAntibody” and “CSTSPProblem.” The “CSTSPAntibody” class inherited from the abstract “CSAntibody” class represents the solutions of the problem. The affinity evaluation function, inputs, and outputs are implemented in the “CSTSPProblem” class inherited from the “ICSOptimizationProblem” interface. Furthermore, to test the customization features of AITSO and to improve the performance of the algorithm, the crossover mechanism, which is a key operation in a genetic algorithm, was introduced to the artificial immune algorithm.

**Figure 10:**
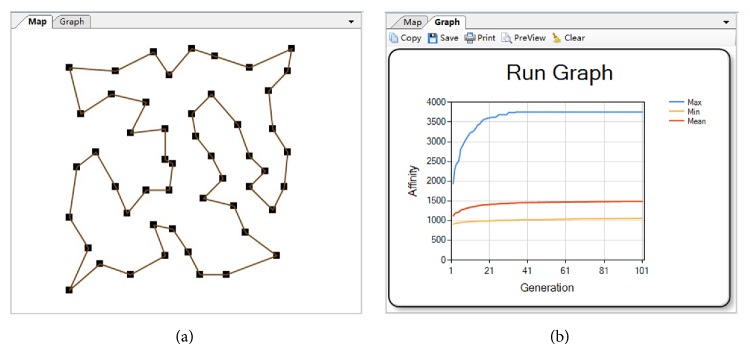
Results of the testing traveling salesman problem.

The TSP dataset used for testing is the Eil51 dataset chosen from the standard TSP library TSPLib95, which has been used as a benchmark testing dataset (download url: http://www.iwr.uni-heidelberg.de/groups/comopt/software/TSPLIB95/tsp/) in many studies. The Eil51 dataset comprises 51 cities, and the best known tour length of the dataset is 429.1822 [39]. The parameters of the artificial immune algorithm were set as follows: the population size was 100 (including 15 memory antibodies), selection ratio was 0.25, cloning coefficient was 0.5, mutation rate was 0.06, crossover rate was 0.4, and the replacement ratio was 0.1. The stopping criterion was that the number of generations exceeds 100. After several experiments, we got the best solution to the problem with a tour length of 428.982 (see Figure 10), a little shorter than the 429.182 mentioned above. These experiments were performed on a MacBook air computer equipped with Intel I5-4250U CPU and 4 GB RAM and the average computation time to solve this problem is 6 seconds.

### 4.2. Optimization of the Design of Environmental Monitoring Networks

An environmental monitoring network optimization problem was employed to demonstrate how to solve environmental modeling and spatial optimization problems in AITSO. The experimental data source code used in this case study can be downloaded from the website https://AITSO.codeplex.com/.

Environmental monitoring networks, composed of several monitoring stations, are used to capture environmental pollution data or quality information of the air, water, and soil of a specific area. The objective in the optimization of an environmental monitoring network is to arrive at the optimal number and spatial layout of the monitoring stations. This is critical to environmental monitoring and assessment since it can help to achieve an optimal configuration of the monitoring stations while obtaining the pollution information accurately under a minimum number of monitoring stations and at least cost [42]. Therefore, the process of designing the network can be seen as a typical spatial optimization problem, and it has been an important research topic in the environmental monitoring community [42–44].

**Figure 11:**
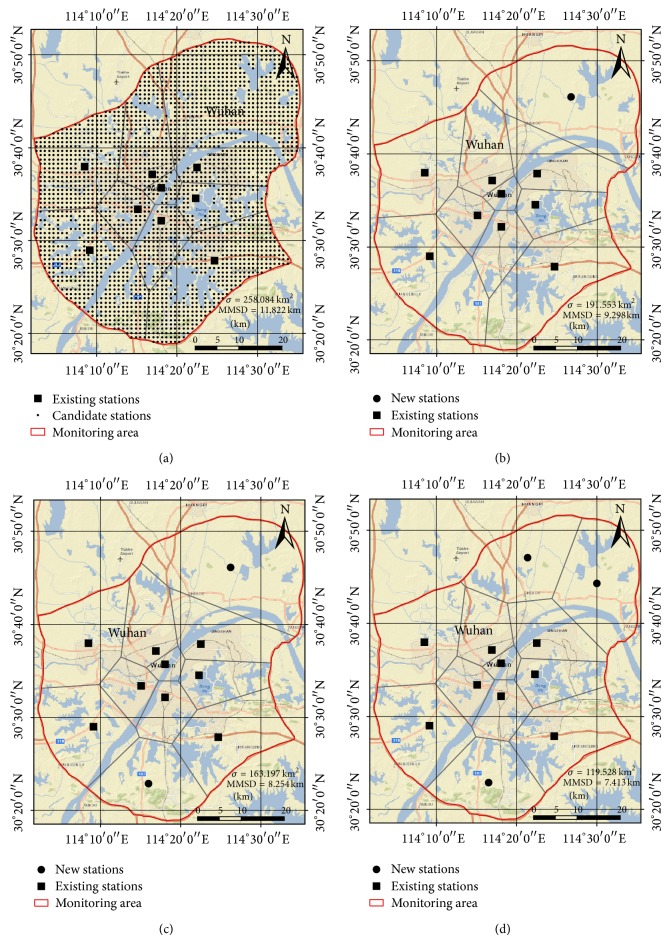
Results of the testing environmental monitoring network optimization problem.

The optimization problem designed for this case was the optimization of the air quality monitoring network of Wuhan, which is located in central China (coordinates: 30°35′N 114°17′E), with a population of over 10 million people (2011 census). As shown in Figure 11(a), the air quality monitoring network of Wuhan is composed of nine monitoring stations. The air quality monitoring network of this city has been in use for several years. However, following the rapid growth of the city, the monitoring area should also have been expanded. In this case study, we assumed that the existing stations remain unchanged, and some new stations need to be added to the existing monitoring network. To solve this problem, the study area was first divided into a grid system in which each grid cell (1 km × 1 km) is a candidate monitoring station. The grid cells which are located in the rivers or lakes were deleted from the candidate station set, and the number of candidate stations was 2529. Therefore, a spatial layout scheme of the new stations was encoded into an antibody in artificial immune system, and a gene of the antibody represents a feasible location of the new stations.

Usually, optimization of the number and spatial layout of the monitoring stations has to deal with some complex influential factors, such as the contamination levels of pollutants, cost and budget, population density, terrain conditions, and spatial coverage. However, the main purpose of this case study was to illustrate the basic principles and methods for developing an optimization application in the AITSO environment and to provide researchers with a simple programming template. Therefore, in this case study, we only considered the spatial coverage of the stations, and the “minimization of the mean of the shortest distances” (MMSD) criterion was adopted as the optimal objective of the problem. MMSD aims to minimize the average of the distances of all grid points to their nearest station [45]. The objective values of the antibodies were calculated by the following formula:

(3) $$\begin{array}{c} \phi_{\text{MMSD}}\left(S\right)=\frac{\sum_{i=0}^{N}d\left(x_{i},S\right)}{N}, \end{array}$$

where *x*_*i*_ is the *i*th grid and *d*(*x*_*i*_, *S*) is the distance between grid *x*_*i*_ and the closest station in the monitoring network. The numbers of new stations were specified in different scenarios (one, two, or three) to simulate the optimization of the monitoring network under different costs and budgets. The optimal monitoring networks are shown in Figures 11(b), 11(c), and 11(d), and the average computation time to solve this application is about 750 seconds. It is believed that the simulation result can help the policy makers to build an optimal monitoring network, according to their budget.

This case describes the general principles and methods of using an immune algorithm to solve an environmental monitoring network optimization problem in AITSO. In this case, only the “MMSD” criterion was considered, and some other important factors were not integrated into the objective functions. However, this case could be extended to optimize different types of environmental monitoring networks (soil, water, disease, etc.) by integrating some important environmental factors, such as the concentration of PM2.5 in the air or the concentration of heavy metals in soil.

## 5. Conclusions and Discussion

Spatial optimization is a type of complicated and widespread problem that is common in spatial planning and geocomputation. The artificial intelligence approaches have provided effective solutions for solving these problems. Therefore, the main contributions of this paper include the following. (i) The characteristics of spatial optimization problems were analyzed and summarized and then a general paradigm for geospatial optimization modelling under the context of geocomputation was proposed in this study. (ii) A unified framework of spatial optimization model was developed to model the geospatial problems in this paper. (iii) The freely accessible tool AITSO, which integrates the artificial immune system algorithms, has been developed as a flexible and extensible tool for researchers and decision makers.

Since the initial testing of the tools demonstrates the potential of AITSO as a flexible tool for the modeling of practical spatial optimization problems such as spatial route optimization and planning and environmental monitoring network optimization, it is therefore believed that AITSO will advance the development and popularity of spatial optimization in spatial optimization modeling and decision making. Benefiting from the loose “plugin host” architecture, developers can easily implement their own problem-specific application plugins or new immune operators based on foundation APIs provided by AITSO to solve practical problems or improve the performance of an algorithm. With these standard interfaces, researchers can easily integrate their own models and knowledge into the tool to solve some special spatial problems. Furthermore, the technologies described in this paper could also help developers to build universal optimization tools based on some other algorithms, such as genetic algorithms, evolution strategies, and particle swarm optimization.

As a universal tool for spatial optimization, the present software still has the following deficiencies, which will be improved in the future: (i) only the clonal selection immune model has been integrated into the tool; some other AIS models such as aiNET have not yet been implemented. Hence, these algorithms will also need to be implemented in the future. (ii) The method used for solving multiobjective problems is now based on a weight-based approach. Since a lot of spatial optimization problems are multiobjective problems, it will be crucial to integrate some more useful multiobjective methods into AITSO, such as the paretodominant strategy. (iii) The process of using artificial intelligence algorithms to solve spatial problems is usually data-intensive or computationally intensive [46]. Therefore, it will be necessary to use parallel computing technology to improve the performance of the algorithm. Parallel programming technology, which is a new feature in  .NET Framework 4, will be used to take advantage of a multicore processor to increase the computational efficiency of the algorithm. Furthermore, we also plan to develop some problem-specific application plugins for solving typical spatial optimization problems such as spatial sampling design and land-use spatial allocation.

## Figures and Tables

**Algorithm 1:**
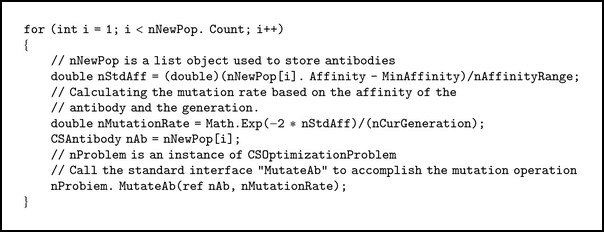
A C# code sample demonstrates how the common operator works.

**Table 1:** Comparison of the popular AI tools for solving spatial optimization problems.

Tools	Programming language	Algorithms	Have GIS functions	Have application programming interfaces	Have graphical user interfaces
GeoSOS	C#	Ant colony optimization	Yes	No	Yes
OAT	Java	Clonal selection algorithms, B-cell algorithm, and so forth	No	Yes	Yes
EO	C++	Evolution algorithms, particle swarm optimization, and so forth	No	Yes	No
GAtool	MATLAB	Genetic algorithms	No	Yes	Yes
